# Prognostic significance of hematological parameters and albumin in BCMA-targeted CAR-T therapy for multiple myeloma

**DOI:** 10.3389/fimmu.2025.1703394

**Published:** 2025-12-17

**Authors:** Liansheng Jiang, Hefei Ren, Zhiqing Ke, Yaoting Liu, Chang Liu, Lin Zhou

**Affiliations:** Department of Laboratory Medicine, Shanghai Changzheng Hospital, Naval Medical University, Shanghai, China

**Keywords:** multiple myeloma, chimeric antigen receptor T, hemoglobin, albumin, refractory or relapsed multiple myeloma, B-cell maturation antigen

## Abstract

Multiple myeloma is an incurable hematologic malignancy, and CAR-T therapy can benefit some patients with relapsed or refractory disease. However, due to individual variations, treatment outcomes vary significantly among different patients. We retrospectively analyzed 77 relapsed/refractory MM patients receiving BCMA-targeted CAR-T therapy. Baseline and dynamic hematological/nutritional parameters were assessed at four time points: prior to CAR-T cell collection, before pre-lymphodepletion therapy, before CAR-T cell infusion, and within the 7-day post-infusion. Prognostic groups were stratified by progression-free survival (PFS ≤ 10 vs. > 10 months). The results demonstrate that patients in the poor prognosis group consistently exhibited significantly lower levels of HGB, RBC, and HCT throughout the treatment period(p<0.05). Before pre-lymphodepletion ALB level in the poor prognosis group was significantly lower than that in the good prognosis group (p<0.05). LDH, creatinine, calcium ions, and β2-microglobulin showed no differences at the four observation time points(p>0.05). ROC analysis confirmed prognostic value for HGB (AUC = 0.693), RBC (AUC = 0.669), HCT (AUC = 0.691), and ALB (AUC = 0.756) (all p < 0.05). Kaplan-Meier analysis linked low HGB (≤92.5 g/L), RBC (≤3.26 ×10¹²/L), HCT (≤32.05%), and ALB (≤35.3 g/L) to inferior PFS (p = 0.011, 0.014, 0.0033, and 0.0001, respectively). Low ALB (≤35.3 g/L) before pre-lymphodepletion is a practical biomarker for risk stratification, reflecting compromised bone marrow reserve and immune-nutritional status. These accessible parameters may optimize patient selection and supportive care strategies.

## 1 Introduction

Multiple myeloma (MM) remains an incurable plasma cell malignancy characterized by clonal proliferation in the bone marrow microenvironment, affecting over 170,000 patients globally annually (1). Despite therapeutic advancements, including proteasome inhibitors and immunomodulatory drugs, a substantial proportion of patients develop refractory or relapsed multiple myeloma (R/RMM) (2). Chimeric antigen receptor T-cell (CAR-T) therapy targeting B-cell maturation antigen (BCMA) has emerged as a breakthrough intervention, demonstrating unprecedented response rates in heavily pretreated R/RMM cohorts (3, 4). However, significant heterogeneity exists in treatment outcomes, with approximately 30-40% of patients experiencing early progression or severe toxicities (5).

Several factors have been examined for their potential to predict response and survival after CAR-T therapy. These include tumor burden, performance status, inflammatory markers (such as ferritin and C-reactive protein), lactate dehydrogenase (LDH) levels, and specific characteristics of the CAR-T product (6). Additionally, the influence of pre-treatment host factors, especially baseline hematological and nutritional parameters, is increasingly acknowledged but less extensively studied. Routine blood parameters, such as complete blood counts (CBC) and serum albumin (ALB), are easily accessible, cost-effective, and reflect the patient’s overall systemic condition, including bone marrow reserve, inflammation, and nutritional status. Growing evidence indicates that anemia and hypoalbuminemia may be linked to poorer outcomes in various cancer treatments (7–11), possibly signaling a compromised physiological state less capable of withstanding intensive therapies or mounting effective anti-tumor immune responses. However, their specific prognostic value within the context of CAR-T therapy, especially throughout the entire treatment course, requires further investigation.

In this study, we conducted a retrospective analysis focusing on readily accessible pre-treatment hematological and biochemical parameters in patients undergoing CAR-T therapy. By evaluating baseline red blood cell indices (RBC count, HGB, HCT) and serum ALB levels—which are readily accessible in standard clinical practice—we seek to identify practical biomarkers that can inform prognosis early in the treatment course. The findings from this investigation have important implications for optimizing patient selection and management in CAR-T therapy. Validation of these easily obtainable baseline parameters may offer clinicians a cost-effective tool for risk assessment, facilitating timely interventions for high-risk patients and promoting more personalized treatment strategies. Importantly, this study underscores the underutilized potential of routine clinical data in advancing precision medicine for cellular immunotherapies, thereby helping to bridge the gap between complex biomarker discovery and real-world clinical application.

## 2 Materials and methods

### 2.1 Patients and samples

The medical charts of 77 MM patients who underwent CAR-T therapy at our institution between December 2018 and April 2023 were analyzed retrospectively. The inclusion criteria were as follows: (a) Meets the diagnostic criteria for MM revised by the International Myeloma Working Group (IMWG) in 2014 (12). (b) Previously received ≥3 lines of systemic therapy, including proteasome inhibitors, immunomodulatory drugs (IMiDs), and CD38-targeted monoclonal antibodies, with documented disease progression or relapse after the most recent treatment regimen, as defined by the International Myeloma Working Group (IMWG) criteria for relapsed and/or refractory multiple myeloma (13). Patients older than 75 years were excluded.

**Figure 1:**
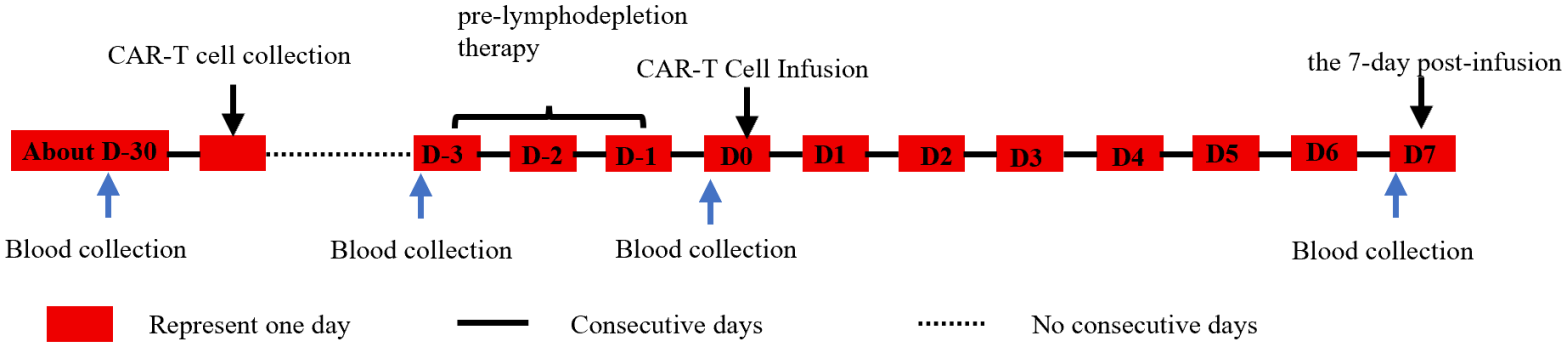
CAR-T cell therapy and blood collection procedure.

The clinical data were retrieved from the hospital’s electronic medical record system. The patients’ comprehensive medical histories were systematically collected, and a panel of baseline laboratory parameters was meticulously recorded, including age, sex, RBC, HGB level, HCT, Ca, LDH, sCr, β2MG, and ALB. The dataset encompasses laboratory test results obtained at four critical time points (**Figure 1**): prior to CAR-T cell collection, before pre-lymphodepletion therapy, before CAR-T cell infusion, and within the 7-day post-infusion. The research project was approved by the Ethics Committee of The Second Affiliated Hospital of Naval Medical University (approval numbers: No. 2021SL034 and No. 2023SLYS7) and was conducted in accordance with the ethical standards outlined in the 1964 Declaration of Helsinki and its subsequent amendments.

### 2.2 Statistical methods

The statistical analyses were conducted utilizing SPSS software (Version 23.0; IBM SPSS Statistics, Chicago, IL, USA), GraphPad Prism (Version 10.5; GraphPad Software, San Diego, CA, USA), and R (Version 4.5.1; R Foundation for Statistical Computing, Vienna, Austria). Categorical variables were analyzed using the chi-square test. The normality of continuous variable distributions was evaluated via the Shapiro-Wilk test. The homogeneity of variances was assessed using Levene’s test. Descriptive statistics for continuous variables were presented as mean ± standard deviation (SD) for normally distributed data or as median with interquartile range (IQR) for non-normally distributed data. Comparative analyses of continuous variables between groups were performed employing either the parametric independent t-test (for normally distributed data) or the nonparametric Mann-Whitney U-test (for non-normally distributed data), contingent upon the normality assessment results. Variables were analyzed using receiver operating characteristic (ROC) curve analysis to predict the outcomes of CAR-T cell therapy by calculating the area under the curve (AUC). Prior to model development, we evaluated multicollinearity between ALB and three hematological parameters (RBC, HGB, and HCT). The results confirmed that all variance inflation factor (VIF) values were below 5 and all tolerance values exceeded 0.1, indicating that multicollinearity would not substantially influence the outcomes. Standard survival analysis methodologies, including Kaplan-Meier survival curves and Gehan-Breslow-Wilcoxon tests, were applied to characterize and compare survival outcomes. *P* < 0.05 is considered statistically significant.

## 3 Results

### 3.1 Patient characteristics

A total of 77 patients with R/RMM who received BCMA-targeted CAR-T therapy at Changzheng Hospital, affiliated with the Naval Medical University (Shanghai, China), between December 2018 and April 2023 were enrolled in the investigation. Based on a completed clinical trial conducted at our hospital, the median PFS was 10 months (14). Accordingly, we stratified patients into two groups: those with PFS >10 months following CAR-T cell therapy were classified as the good prognosis group(n=56), while those with PFS ≤10 months were designated as the poor prognosis group(n=21). The clinical characteristics and treatment regimens of the two patient cohorts are summarized in **Table 1**.

**Table 1:** Comparative analysis of continuous variables in multiple myeloma patients following CAR-T cell therapy.

Variables	Good prognosis group	Poor prognosis group	p
Sex (Male vs. Female)	28/28	9/12	0.617
Age (Year)	61.11 ± 8.02	58.19 ± 7.73	0.156
DS stage (III vs. II/I)	56/0	21/0	–
ISS stage (III vs. II/I)	20/36	6/15	0.601
Prior ASCT, n (%)	17(30.4)	11(52.4%)	–
Prior PI, n (%)	54(96.4%)	21(100%)	–
Prior IMiD, n (%)	54(96.4%)	20(95.2%)	–
Prior mAb, n (%)	10(17.9%)	7(33.3%)	–
Prior Che, n (%)	34(60.7%)	16(76.2%)	–

*DS stage*, Durie-Salmon stage; *ISS, stage* International Staging System; *ASCT*, Autologous Stem Cell Transplantation; *PI*, Proteasome Inhibitors; *IMiD*, Immunomodulatory agents; *mAb* Monoclonal Antibody; *Che*, Cytotoxic Chemotherapy.

### 3.2 Laboratory characteristics

Comparative analysis of continuous variables across the good and poor prognosis groups at four distinct time points (**Table 2**). Throughout the entire treatment course, the poor prognosis group consistently demonstrated significantly lower levels of hematological parameters. Prior to CAR-T cell collection, the poor prognosis group exhibited markedly reduced HGB levels (91.19 ± 24.20 g/L vs. 106.73 ± 18.03 g/L, p=0.003), RBC count (2.91 ± 0.86 ×10¹²/L vs. 3.39 ± 0.64 ×10¹²/L, p=0.009), and HCT values (28.45 ± 6.82% vs. 32.90 ± 5.11%, p=0.003) compared to the good prognosis group. At the start of the pre-lymphodepleting therapy, the ALB level in the poor prognosis group was significantly lower than in the good prognosis group (34.04 ± 5.61 vs. 38.93 ± 5.51, p=0.001), and this difference remained throughout the subsequent treatment process. LDH, creatinine, calcium ions, and β2-microglobulin showed no differences at the four observation time points (p>0.05).

**Table 2:** Comparative analysis of continuous variables across favorable and unfavorable prognosis groups at distinct time points.

Indicators	Good Prognosis Group	Poor Prognosis Group	P
Prior to CAR-T Cell Collection			
RBC (×10¹²/L)	3.39 ± 0.64	2.91 ± 0.86	0.009
HGB(g/L)	106.73 ± 18.03	91.19 ± 24.20	0.003
HCT (%)	32.90 ± 5.11	28.45 ± 6.82	0.003
ALB(g/L)	38.07 ± 5.68	36.81 ± 4.96	0.373
Ca (mmol/L)	2.20 ± 0.16	2.23 ± 0.22	0.459
sCr(mmol/L)	72.25 ± 24.35	62.00 ± 23.86	0.065
β2MG ^*^ (mg/L)	2.71[2.01,3.80]	2.69[2.29,3.59]	0.970
LDH^*^ (U/L)	181[150,225]	229 [129,325]	0.543
Before Pre-lymphodepletion Therapy			
RBC(×10¹²/L)	3.41 ± 0.77	2.83 ± 0.70	0.004
HGB(g/L)	105.98 ± 21.71	89.33 ± 22.14	0.004
HCT(%)	32.52 ± 6.27	28.03 ± 6.32	0.007
ALB(g/L)	38.93 ± 5.51	34.04 ± 5.61	0.001
Ca(mmol/L)	2.19 ± 0.19	2.21 ± 0.10	0.697
sCr^*^ (mmol/L)	69[57.5,84.5]	62.5[48.5,72.75]	0.170
β2MG^*^ (mg/L)	2.46[2.16,3.51]	3.30[2.41,4.45]	0.157
LDH^*^ (U/L)	171[149.5,191.5]	170[147.5,265.5]	0.121
Before CAR-T Cell Infusion			
RBC (×10¹²/L)	3.11 ± 0.71	2.57 ± 0.68	0.004
HGB(g/L)	97.00 ± 19.85	81.19 ± 20.58	0.003
HCT(%)	29.61 ± 5.75	25.26 ± 6.07	0.005
ALB(g/L)	35.24 ± 3.80	32.19 ± 4.56	0.004
Ca(mmol/L)	2.11 ± 0.13	2.17 ± 0.13	0.062
sCr^*^ (mmol/L)	60[46.5,72.5]	55.5[41.0,67.3]	0.288
β2MG^*^ (mg/L)	2.53[1.99,3.25]	2.92[2.08,3.87]	0.512
LDH^*^ (U/L)	164[147,190.5]	163[142.5,232]	0.296
7-day Post-infusion			
RBC (×10¹²/L)	3.03 ± 0.72	2.59 ± 0.65	0.018
HGB(g/L)	95.39 ± 20.09	81.86 ± 19.74	0.010
HCT(%)	29.07 ± 5.79	25.27 ± 5.89	0.013
ALB(g/L)	35.08 ± 3.67	32.71 ± 4.97	0.026
Ca(mmol/L)	2.09 ± 0.15	2.09 ± 0.14	0.978
sCr^*^ (mmol/L)	63[55.5,81.5]	59[50.3,71.0]	0.151
β2MG^*^ (mg/L)	2.46[2.05,3.16]	3.09[2.46,5.02]	0.076
LDH^*^ (U/L)	186[162,186]	240.5[153,380.5]	0.104

^*^ The data don’t follow a normal distribution and are presented as median with interquartile range (IQR). RBC Red Blood Cell; HGB hemoglobin; HCT Hematocrit; LDH Lactate Dehydrogenase, ALB Albumin, sCr serum Creatinine, Ca Calcium ion, β2MG β2-Microglobulin.

### 3.3 ROC curves of RBC, hemoglobin level, hematocrit, and ALB

**Figure 2:**
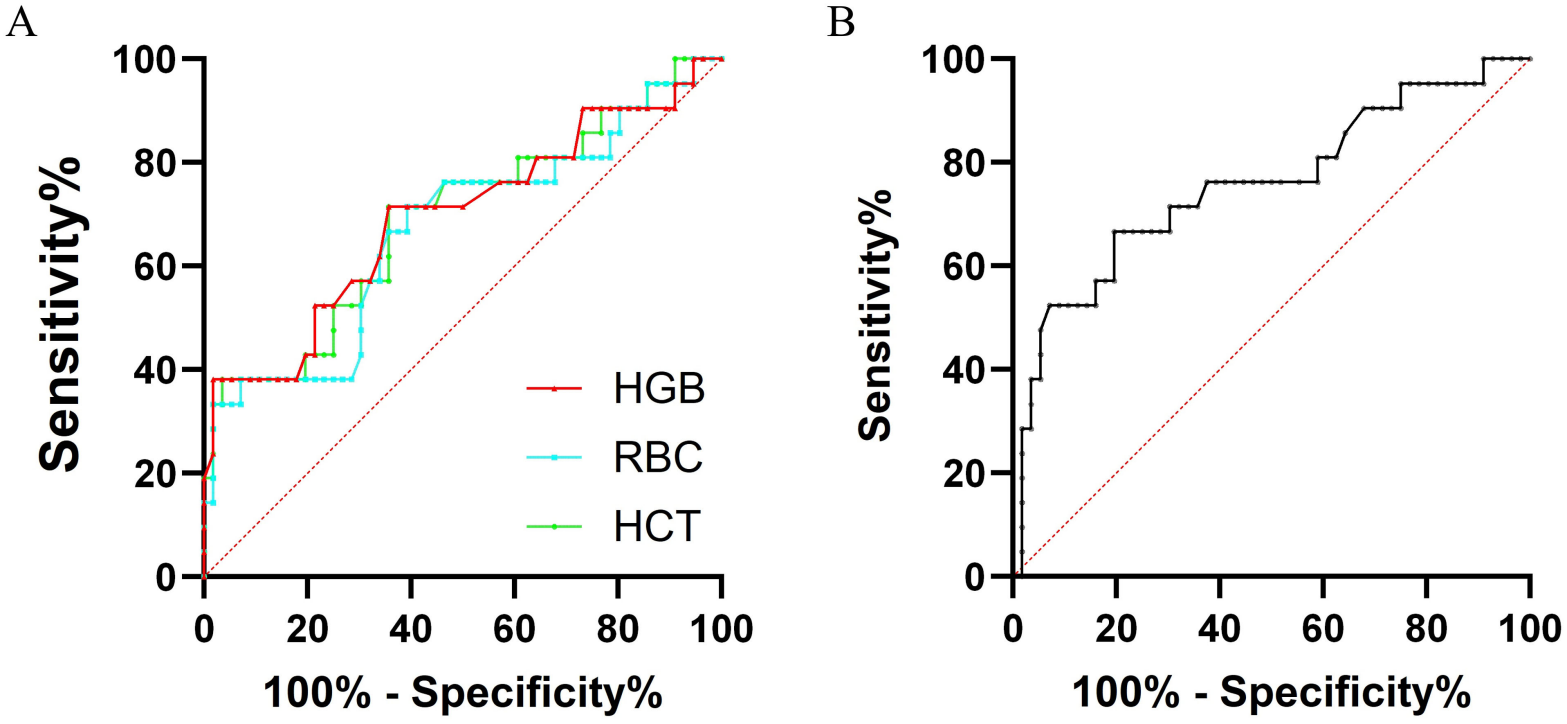
ROC curve analyses in the prognostic value of CAR-T therapy. **(A)** ROC curves of RBC, Hemoglobin and HCT: RBC, AUC 0.669, 95% CI 0.5228-0.8148, P < 0.05; Hemoglobin, AUC 0.693, 95% CI 0.5486-0.8375, P < 0.01; HCT, AUC 0.691, 95% CI 0.5492-0.8326, P < 0.05; **(B)** ROC curve of pre-lymphodepletion ALB levels, AUC 0.756, 95% CI 0.6243-0.8885, P < 0.001. ROC, receiver operator characteristics; RBC, Red Blood Cell; HGB, hemoglobin; HCT, Hematocrit; ALB, Albumin; AUC, area under the curve; CI, confidence interval.

To further evaluate the prognostic value of pre-CAR-T cell collection RBC count, HGB level, and hematocrit, as well as the prognostic significance of pre-lymphodepletion ALB levels, we conducted ROC curve analyses for these biomarkers. The AUCs for RBC, HGB level, and HCT as predictors of CAR-T therapy success were 0.669 (95% confidence interval [CI]: 0.5228-0.8148, p < 0.05), 0.693 (95% CI: 0.5486-0.8375, p < 0.01), and 0.691 (95% CI: 0.5492-0.8326, p < 0.05), respectively. Additionally, the AUC of ALB was 0.756 (95% CI: 0.6243-0.8885, p < 0.001) (**Table 3**; **Figure 2**).

**Table 3:** ROC curve analyses for evaluating the prognostic value of CAR-T therapy.

Indicators	AUC	Cutoff value	Sensitivity%	Specificity%	p
RBC	0.669	3.26	71.43	60.71	0.0232
HGB	0.693	92.5	52.38	78.57	0.0094
HCT	0.691	32.05	71.43	64.29	0.0102
ALB	0.756	35.3	66.67	80.36	0.0006

RBC, Red Blood Cell; HGB, hemoglobin; HCT, Hematocrit.

### 3.4 RBC, HGB, HCT, and ALB with clinical outcomes

**Figure 3:**
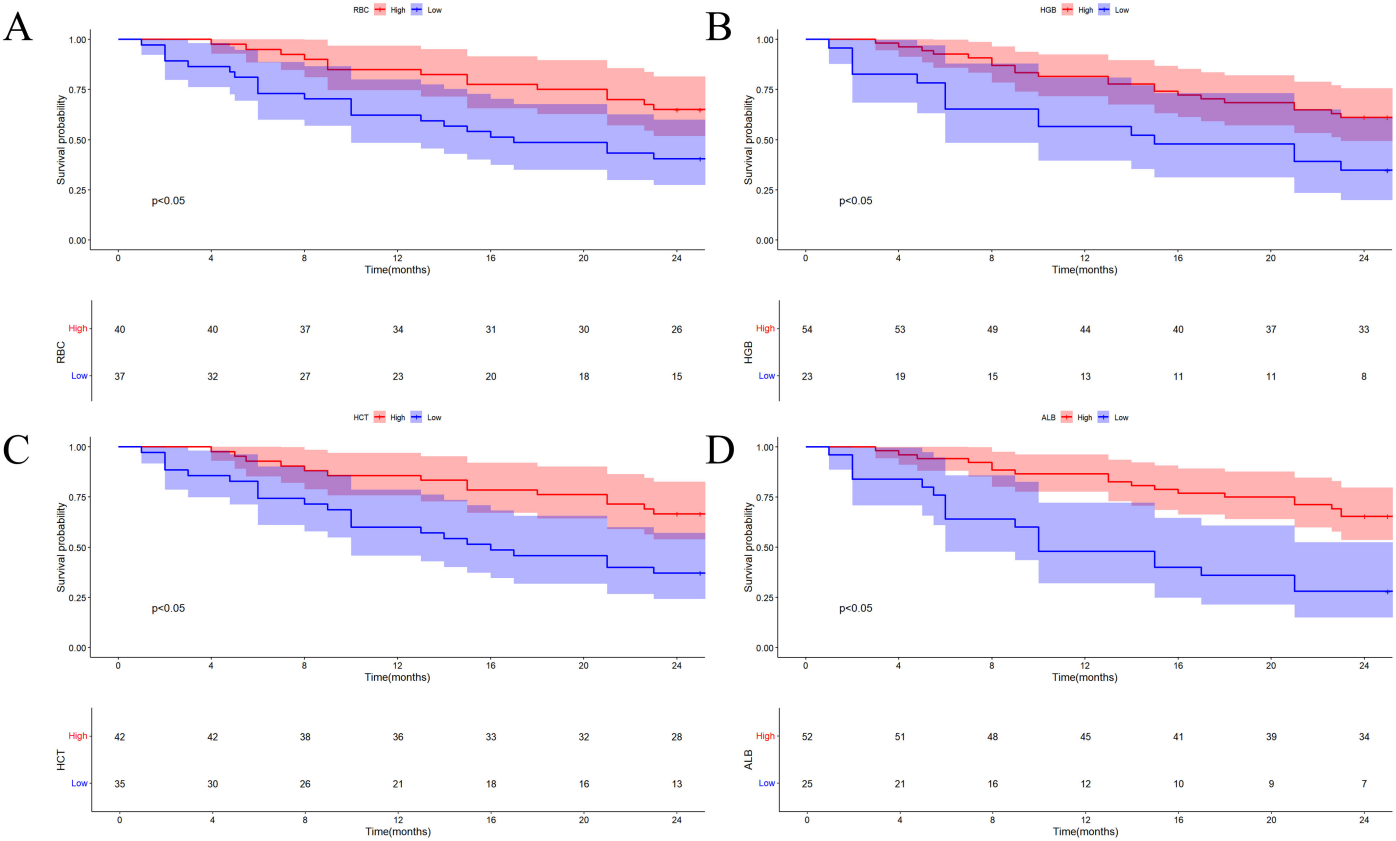
Kaplan-Meier survival curves (univariate analysis) were analyzed using the Gehan-Breslow-Wilcoxon test for progression-free survival (PFS) according to levels of RBC **(A)**, HGB **(B)**, HCT **(C)**, and ALB **(D)**. RBC, Red Blood Cell. HGB, hemoglobin. HCT, Hematocrit. ALB, Albumin. PFS: progression-free survival.

The clinical laboratory parameters collected before CAR-T cell apheresis are vital criteria for assessing whether to proceed with CAR-T cell therapy. Therefore, we analyzed the correlation between patients’ pre-collection RBC counts, HGB levels, and HCT, and their PFS. Similarly, we examined the relationship between pre-lymphodepletion therapy ALB levels and PFS in patients. The cutoff value for each indicator’s expression level serves as the boundary for dividing patients into high- and low-expression groups. Low levels of RBC, HGB, HCT, and ALB are linked to poorer PFS (p=0.014, p=0.011, p=0.0033, p=0.0001, respectively) (**Figure 3**).

## 4 Discussion

CAR-T cell therapy demonstrates high response rates in R/R MM, yet significant variations exist in patient prognosis (6). Early assessment of prognostic factors enables proactive development of supportive care plans, thereby reducing treatment-related risks. This study conducted a retrospective analysis of data from 77 patients with relapsed/refractory MM who received CAR-T therapy, systematically reporting the correlation between dynamic changes in RBC parameters (RBC counts, HGB, HCT) and ALB throughout the treatment course and patient prognosis. We observed that low levels of RBC, HGB, and HCT prior to CAR-T therapy were associated with patients in the poor prognosis group, and this difference persisted throughout the entire treatment process. (**Table 2**). Pre-lymphodepletion dissection ALB (cut-off value 35.3 g/L) was identified as the predictive indicator (AUC = 0.756, P<0.001), patients in the low ALB group demonstrated significantly shorter progression-free survival (PFS) (P = 0.0001).

The underlying anemic state may reflect the degree of bone marrow microenvironment exhaustion. The bone marrow infiltration in MM directly suppresses erythropoiesis. A reduction in RBCs may impair oxygen delivery and T-cell metabolic efficiency, which aligns with the theory that CAR-T efficacy requires a functional immune microenvironment. Hematopoietic suppression represents a frequently encountered adverse effect of CAR-T cell therapy, with particularly pronounced severity observed in patients suffering from R/RMM. Maria Luisa Palacios-Berraquero et al. (15) co-cultured the supernatant from CAR-T cells and MM cells with bone marrow mononuclear cells from healthy donors, followed by single-cell sequencing. The results showed that early hematopoietic differentiation transcription factors (GATA2, RUNX1, CEBPA) were upregulated under the influence of the CAR-T supernatant, while the activity of key regulators of neutrophil and monocyte maturation (ID2 and MAFB) was reduced. Erythroid progenitor cells also exhibited negative regulation of the G2/M phase and mitotic cell cycle transition, suggesting a decreased proliferation rate. Therefore, we speculate that under the stimulation of CAR-T cells, the patient’s originally poor hematopoietic environment becomes even more challenging. This dysfunctional hematopoietic state subsequently leads to insufficient energy and oxygen supply for CAR-T cells, ultimately contributing to treatment failure.

Albumin, the predominant plasma protein, functions as an important indicator for assessing the nutritional status and prognosis of patients undergoing CAR-T therapy. The study conducted by Cucchiaro et al. (16) identified serum ALB level as an independent negative prognostic factor for 12-month overall survival (OS) in patients with B-cell non-Hodgkin lymphoma (NHL) or B-cell acute lymphoblastic leukemia (ALL) receiving CAR-T cell therapy. Additionally, another investigation revealed that reduced serum ALB concentrations function as predictive biomarkers for both the toxicity profile and clinical outcomes in MM patients undergoing anti-BCMA CAR-T cell therapy (17). The findings of this study are similar to our conclusions. Several studies have demonstrated that serum ALB levels are significantly correlated with the incidence of adverse reactions following CAR-T cell therapy. Shuyi Ding et al. (18) revealed a significant negative correlation between serum ALB levels on day 7 post-CAR-T infusion and the severity of cytokine release syndrome (CRS). Additionally, in a separate study of patients with non-Hodgkin lymphoma (NHL) receiving CD19-targeted CAR-T cell therapy, lower pretreatment serum ALB levels were associated with an elevated risk of acute kidney injury (AKI). Notably, patients who developed AKI following CAR-T therapy exhibited significantly reduced overall survival rates compared to those without AKI (19). The emergence of these complications may have contributed to the failure of CAR-T therapy.

Based on our research findings, we hypothesize that implementing nutritional interventions and optimizing hematopoietic function prior to CAR-T cell therapy may constitute highly effective strategies for enhancing treatment outcomes. Consequently, the validity of this hypothesis can be further validated in clinical practice through the application of targeted interventions, followed by systematic evaluation and comparative analysis of variations in patient outcomes.

The limitations of this study are chiefly due to its single-center, retrospective design, which may introduce selection bias. The potential variations in patient demographics, clinical protocols, or healthcare environments necessitate validation of these findings through multicenter investigations to ensure their generalizability to broader populations. Additionally, the retrospective nature of the study inherently risks information bias, as data acquisition relies on existing medical records that may be incomplete or inconsistently documented. Future research should prioritize multicenter, prospective study designs with standardized protocols to substantiate these findings and enhance their external validity.

## 5 Conclusion

Red blood cell parameters (RBC counts, HGB, HCT) and ALB are very useful indicators for predicting the outcome of CAR-T therapy in patients with MM.

## Data Availability

The raw data supporting the conclusions of this article will be made available by the authors, without undue reservation.
